# Smartphone-based photogrammetry and videogrammetry versus structured light scanning for the assessment of facial landmark distances and uncertainty

**DOI:** 10.1007/s00414-026-03921-y

**Published:** 2026-07-11

**Authors:** Luiz Carlos Teixeira Coelho, Flávia Martinez de Carvalho, Luiz Felipe de Almeida Furtado, Luis Lerma-Doménech, Matheus Ferreira Coelho Pinho, Francisco Airasca Altónaga, Samir de Souza Oliveira Alves, Ana Luiza Meneguci Moreira Franco, José Luis Lerma

**Affiliations:** 1 Photogrammetry and Remote Sensing Laboratory (Laboratório de Fotogrametria e Sensoriamento Remoto—LFSR), School of Engineering, Rio de Janeiro State University, Rua São Francisco Xavier 524, PJLF Sala 4044F, Maracanã, Rio de Janeiro, RJ 20550-013 Brazil; 2 Photogrammetry and Laser Scanner Research Group (GIFLE), Department of Cartographic Engineering, Geodesy and Photogrammetry, Universitat Politècnica de València, Camino de Vera s/n, Valencia, 46022 Spain; 3 Laboratory of Epidemiology of Congenital Malformations (LEMC—Laboratório de Epidemiologia das Malformações Congênitas), Instituto Oswaldo Cruz, Avenida Brasil 4365 LEMC, Manguinhos, Rio de Janeiro, RJ 21040-360 Brazil; 4 Post-Graduate Programme in Biological Sciences (Genetics), Federal University of Rio de Janeiro, Rua Professor Rodolpho Paulo Rocco, s/n, Prédio do CCS-Bloco A-2, Andar-Sala 099, Cidade Universitária, Rio de Janeiro, RJ 21941-617 Brazil; 5 Faculty of Medicine and Dentistry, Universitat de València, Av. de Francisco Tomás y Valiente, 2, Valencia, 46010 Spain; 6 Post-Graduate Program in Computational Sciences and Mathematical Modeling, Institute of Mathematics and Statistics, Rio de Janeiro State University, Rua São Francisco Xavier, 524 PJLF Sala 6007, Maracanã, Rio de Janeiro, 20.550-900 Brazil

**Keywords:** Photogrammetry, Videogrammetry, Facial landmarks, Structured light scanning, Measurement, Compatibility

## Abstract

**Purpose:**

This study evaluates the reliability of three-dimensional (3D) facial measurements obtained from different acquisition technologies. Specifically, it investigates whether low-cost smartphone-based photogrammetry and videogrammetry can produce measurements comparable to those derived from structured light scanning, and identifies sources of variability associated with each modality.

**Methods:**

Fifteen participants were imaged using three techniques: structured light scanning, smartphone photogrammetry, and smartphone videogrammetry. Inter-landmark distances were computed for each resulting 3D model, including both stable anatomical landmarks and those prone to placement difficulty. A statistical workflow was applied, incorporating non-parametric hypothesis testing, iterative outlier detection, and normality assessments to evaluate measurement variability attributable to acquisition modality, subject movement, and landmark uncertainty.

**Results:**

The three acquisition modalities produced broadly comparable inter-landmark measurements, indicating no substantial loss of accuracy when using smartphone-based methods. However, increased variability was observed in anatomically complex regions, particularly the perioral area, as well as in landmarks known to be difficult to localize. These discrepancies persisted across all modalities, including structured light scanning.

**Conclusions:**

Smartphone-based photogrammetry and videogrammetry represent viable, low-cost alternatives to structured light scanning for 3D facial measurement. Nonetheless, landmark-specific uncertainty remains a critical factor, particularly in challenging anatomical regions. The findings highlight the importance of considering both acquisition modality and landmark reliability in the design and reporting of facial anthropometric studies.

## Introduction

Facial asymmetry is a common feature of human morphology, shaped by a complex interplay of genetic, environmental, and health-related factors. It arises from the interaction of multiple biological processes [1]. Two well-characterized forms of asymmetry are directional asymmetry, which reflects consistent left–right differences (e.g., organ placement), and fluctuating asymmetry, which refers to subtle, random deviations that emerge during embryonic development [1–3]. Accurate detection and quantification of facial asymmetries are essential for a wide range of applications, including clinical diagnostics, treatment planning, anthropological research, and aesthetic evaluations [4]. Over time, various methodologies have been developed to assess facial asymmetry, with increasing emphasis on precision, reproducibility, and objectivity.

Facial landmarks provide a critical spatial framework for analysing symmetry and detecting morphological deviations. By comparing the relative positions of these landmarks, researchers can identify subtle asymmetries and monitor changes over time [5]. However, the reliability of measurements derived from these landmarks varies considerably, influenced by their anatomical definition, visibility, and consistency across observers, algorithms, and reconstruction methods [6, 7].

Historically, facial measurements relied on two-dimensional (2D) photographs, but these are limited by perspective distortion and lack of depth information, compromising reliability [8]. The advent of three-dimensional (3D) imaging technologies, such as stereophotogrammetry and structured light scanning, has transformed the way facial morphometric analysis is carried out nowadays. State-of-the-art technologies generate high-resolution 3D models, enabling more precise landmark identification and significantly improving the quantification of shape variation and asymmetry [4, 9].

### 3D reconstruction methods for facial asymmetry detection

Close-range photogrammetry, operating at distances from 30 cm to 1 m [10], has become a critical tool across numerous fields, including biomedical medicine and forensic sciences [11–14]. Its growth is boosted by low-cost sensors and advanced algorithms, allowing detailed reconstruction of complex surfaces such as the human face [15]. The process involves capturing multiple overlapping images from different viewpoints. Through stages such as alignment, dense cloud creation, and meshing, these images are processed to generate accurate 3D models [13, 16]. The success of this method depends on both the equipment and the operator’s skill in data acquisition and processing.

Videogrammetry adapts this approach by using video frames rather than static images, which is well-suited for capturing rapidly changing objects [17]. A key practical advantage is the reduced acquisition time [18], making it a faster alternative for 3D head reconstruction. Successful implementation requires careful sensor movement during video capture to ensure adequate surface coverage and frame overlapping, though this can result in large data volumes that require strategic frame selection during post-processing [19].

Structured light scanning is renowned for its high precision. This technique projects a known light pattern onto an object and uses a camera to capture the pattern’s deformation. Based on triangulation, the system calculates the 3D geometry of the surface [20]. While it is fast, accurate, and reproducible, this method can be sensitive to ambient lighting conditions, which may interfere with the projected pattern and distort the scan [21].

Comparative studies have thoroughly evaluated the precision of these methods for facial assessment. A systematic review concluded that while stationary stereophotogrammetry systems achieve the highest accuracy, all major devices—including smartphone-based systems—demonstrate sufficient precision for clinical use [15]. This is supported by multiple studies showing that measurements from 3D models, whether created via photogrammetry or structured light, show high agreement with direct calliper measurements, with mean differences often at millimetre level [22, 23].

Research consistently shows the superiority of 3D methods over 2D photography, which exhibits statistically significant differences and low reliability for clinical measurements [24]. In contrast, a metric evaluation of a skull model found that photogrammetry produced results with a minimal mean distance (0.41 mm) compared to a high-accuracy 3D scanner, despite some discrepancies in complex areas such as the eye orbits [25]. These collective findings demonstrate that close-range photogrammetry and videogrammetry may provide a reliable basis for facial measurements and asymmetry detection, effectively supporting their applications in clinical and anthropometric research. Overall, while structured light remains the most precise technique, close-range photogrammetry and videogrammetry have reached accuracy levels suitable for clinical and anthropometric applications, particularly when optimised workflows and stable acquisition conditions are ensured.

However, despite the proven capabilities of these methods, further investigation is needed to determine whether standard cell-phone imagery, given its ubiquity and low cost, can achieve reconstructions comparable to those obtained with multi-camera arrays or structured-light systems. If validated, such an approach would represent a significant step toward democratizing healthcare by lowering the technological barriers to precise facial analysis. In resource-limited settings, where access to specialized equipment is often prohibitive, harnessing widely available smartphone technology could make high-quality craniofacial assessment more equitable, enabling earlier diagnosis and treatment planning in lower‑middle‑income countries without compromising on clinical rigor.

### Objectives of this study

While 3D scanning, photogrammetry, and videogrammetry are established methods for quantifying facial asymmetry, the accessibility and cost-effectiveness of smartphone-based solutions present a compelling alternative [26]. This study investigates whether low-cost photogrammetry and videogrammetry, using cell-phone cameras, can achieve measurements comparable to those obtained with a structured-light scanner, the current gold standard in clinical 3D imaging.

Also noteworthy is the difficulty in discerning whether observed errors stem from the methodologies or from subtle, inherent facial movements [27] that create variability between 3D captures. This distinction is critical, as the growing reliance on 3D models for asymmetry measurements means that such variations directly affect the accuracy and precision of landmark positioning. This inherent variability poses a significant challenge for high-precision applications, as it conflates methodological noise with the true anatomical signal.

In this regard [28], evaluated the impact of involuntary facial movements on the accuracy of measurements in a 3D model generated using a system that combines stereophotogrammetry and structured light. Although the system demonstrated high technical precision (mean error of 0.09 mm), participants’ involuntary facial movements introduced an additional error of 0.32 mm, increasing the overall mean error to 0.41 mm. These measurements were applied to the whole model, without distinguishing specific landmarks that are more prone to error. Consequently, some anthropometric landmarks require a more detailed revision.

To evaluate the applicability of low-cost photogrammetry and videogrammetry for facial asymmetry detection, and the inherent quality of certain landmarks, a dataset of fifteen patient faces was generated using three distinct technologies (structured-light scanning, smartphone-based photogrammetry, and smartphone-based videogrammetry), yielding a total of forty-five 3D facial meshes. By comparing inter-landmark distances across these capture methods, this study seeks (1) to validate whether cell-phone imagery and video frames can produce reliable 3D meshes for facial asymmetry measurements, and (2) to identify which facial landmarks consistently yield dependable metric data and which are prone to greater measurement uncertainty. Accordingly, the primary objective is to establish a foundational methodology for exploring low-cost alternatives that, under specific clinical or research scenarios, can serve as viable options for facial asymmetry assessment.

## Materials and methods

### Patients

Fifteen volunteers were recruited to act as prospective patients for testing image-based facial assessment methods. The cohort’s demographic composition was as follows: nine were of European origin, five were South American, and one was from China. Ethnically, the group included eleven Caucasian, three Mixed-race individuals, and one Asian individual. Participant ages ranged from 22 to 43 years, with a median age of 24. Seven patients were women and eight were men. Informed consent was obtained from all subjects involved in the study, which included 3D scans, photographs and videos of individuals’ faces and of the procedure.

### Materials

#### Sensors

For photogrammetric tasks, a Samsung Galaxy S22 smartphone was employed, with images captured at maximum resolution (3000 × 4000 pixels), and video images (1920 × 1080 pixels) at 60 Hz. This sensor has previously been validated; the image quality is sufficiently precise for photogrammetry [29, 30] and videogrammetry [27].

Additionally, a full facial scan was acquired for each patient using a portable white light scanner. The scanner used for this study was the ACADEMIA 50 scanner by Creaform Inc. (Lévis, Québec, Canada), a professional-grade, portable 3D digitisation tool. This device, which utilises white light technology to achieve high precision, is widely employed across various engineering sectors. Its technical specifications include an accuracy threshold of 0.250 mm and a resolution of 0.250 mm [31]. The scanner was operated via the VXElements software platform, which provides features such as self-positioning with targets, integration of geometry and texture, and enhanced precision through contour optimization [32]. Following calibration, the scanner generated a textured 3D mesh for each patient, leveraging its integrated camera for texture mapping.

#### Orientation marks

A combination of a coded cap and small adhesive markers was used to establish a set of reference points on the subject’s face. This setup, based on the one developed for PhotoMeDAS [33]. Each marker features a unique, non-repetitive pattern, creating a structured framework that enables the software to accurately recognise and align images during data processing [34]. Additionally, circular retro-reflective markers used for 3D scanner orientation were placed on the cap and face.

#### Software employed for photogrammetric processing and 3D mesh manipulation

Agisoft Metashape 1.7 (Agisoft LLC) is a widely-used software for generating 3D spatial data through photogrammetric processing of digital images. It is distinguished from similar applications by its capacity to efficiently process large datasets [35]. The software employs the robust Structure from Motion and Multi-View Stereo (SfM/MVS) algorithm [36], and both images and video frames were processed identically using a standard photogrammetric workflow.

The final output was a textured 3D mesh from the camera motion, plus a 3D mesh obtained from the white light scanner output. For measurements between facial landmarks on the mesh, MeshLab [37] was used. Developed by ISTI - CNR, MeshLab is a free, open-source software employed for all mesh processing and management operations.

### Methodology

The methodological workflow was structured into three sequential phases:

- Data Acquisition: Facial data were acquired through a 3D scan of each participant, supplemented by a series of photographs and video recording.
- Data Processing: The acquired images and video frames underwent photogrammetric processing to generate 3D meshes. These meshes were then trimmed, registered, and used to extract individual measurements.
- Statistical Analysis: A comparative analysis of measurements between anatomical landmarks was conducted across all methods, utilising statistical coefficients and hypothesis testing.

#### Data acquisition

**Fig. 1:**
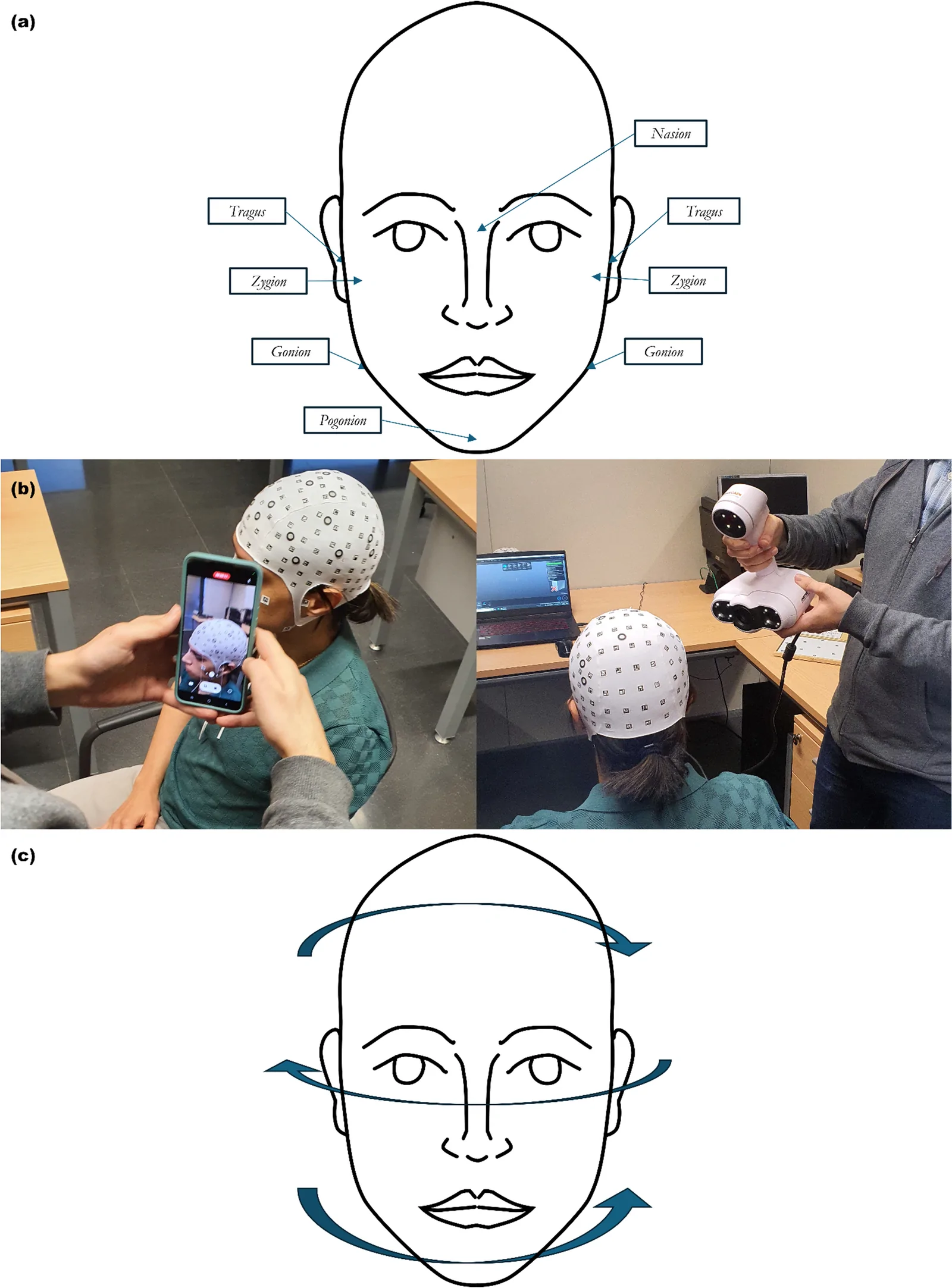
(**a**) Placement of coded stickers on key anatomical landmarks. (**b**) Simultaneous data acquisition using a mobile phone and a white light scanner. (**c**) The standardised movement pattern was performed by participants during all photo/video acquisitions and face scans

The fifteen aforementioned patients participated after giving their informed consent. Each was instructed to wear a PhotoMeDAS coded cap and had eight uniquely coded stickers attached to their face, with five additional stickers placed at various locations (Fig. 1(a)).

The five anatomical landmarks were defined as follows, in accordance with [6] and [38], without considering their dynamic variability:

- Nasion: The midline point on the skull where the frontal and nasal bones intersect.
- Tragus: The cartilaginous projection anterior to the ear canal.
- Zygion: The most lateral point of the zygomatic arch.
- Gonion: The most lateral and inferior point on the mandibular angle.
- Pogonion: The most anterior projecting point of the mandible.

Those five additional facial stickers served for some purposes. On one hand, they ensured more robust photogrammetric adjustment by covering the surface edges, and provided identifiable features in smooth, low-contrast skin regions. Additional markers on the cap itself enhanced the 3D scanner’s alignment and mesh quality, while also acted as high-contrast points for photogrammetry. Also, since areas such as lips and eyes naturally offer high contrast, the combination of coded cap, markers, and natural landmarks was deemed sufficient for reliable processing. Finally, more facial landmarks were provided for the measurement analysis.

For data acquisition, each patient remained seated and still. The procedure began with a face and neck scan using a calibrated Academia 50 3D scanner. This was followed by smartphone photos taken from various angles, and finally, a single, continuous video following a predetermined path around the patient (Fig. 1 (b)).

A consistent imaging protocol ensured data acquisition. For each subject, a minimum of forty-five photos were taken along three semicircular paths: around the upper head, mid-head, and jaw/neck (Fig. 1 (c)). The video followed the same trajectory without interruption. All media were captured at the phone’s maximum resolution (4000 × 3000 for photos; 1920 × 1080 for video). For video processing, frames were extracted at 3 fps.

The scanning procedure was successful for all 15 patients, with scans taking 4–7 min for 13 of them. Two cases required additional time: Patient 11’s beard interfered with the scanner, and Patient 8’s involuntary movements introduced artefacts and blurred some meshes despite multiple scans. In both cases, the affected regions of the meshes did not include the landmarks used in this study, which remained visible in all models. Accordingly, all 45 meshes (15 patients × three methods) were retained. Table 1 summarizes the results of the data collection.

**Table 1:** Data collection for patients with the ACADEMIA 50 3D scanner, and the Galaxy Samsung S22 smartphone in camera and video mode

Patient	3D scanning time	Photogrammetry time	Number of images	Videogrammetry time	Number of video frames
1	5 min	1 min	52	48 s	144
2	6 min	1 min	51	52 s	156
3	5 min	1 min	50	49 s	147
4	7 min	1 min	48	57 s	171
5	5 min	1 min	47	47 s	141
6	4 min	1 min	51	47 s	141
7	6 min	1 min	47	49 s	147
8	13 min	1 min	56	45 s	135
9	7 min	1 min	65	59 s	177
10	5 min	1 min	53	35 s	105
11	11 min	1 min	50	63 s	189
12	5 min	1 min	47	47 s	141
13	4 min	1 min	48	47 s	141
14	6 min	1 min	47	45 s	135
15	5 min	1 min	53	61 s	183

#### Photogrammetric processing and 3D mesh measurements

Both photogrammetric images and video frames were processed. The workflow consisted of:´.

- Alignment: images were aligned with “Generic Preselection” at medium quality.
- Dense cloud generation: a dense point cloud was built at medium quality, limited to one million points per image and filtered for confidence.
- Mesh generation: A high-quality, non-textured 3D mesh was created.

This methodology was not designed to maximize reconstruction quality. Instead, the objective was to reproduce a realistic low-cost workflow that could be implemented by institutions with limited computational resources. For this reason, a medium-quality processing setting was selected rather than the highest available option. Furthermore, one of the long-term goals of this research is to adapt the workflow for mobile applications with cloud-based processing, where computational efficiency and data transfer requirements are critical considerations. Consequently, the number of reconstructed points was intentionally limited (approximately one million points per model) to maintain a practical balance between model quality and processing demands.

Additionally, preliminary tests were conducted using higher-quality reconstruction settings. However, these tests did not produce meaningful improvements in the anthropometric measurements or overall geometric characteristics of the resulting models when compared with those generated using the selected medium-quality configuration. Given the absence of substantial gains in accuracy, and considering the significantly longer processing times associated with higher-quality settings, the present study focuses on the configuration that achieved comparable results while remaining computationally efficient.

**Fig. 2:**
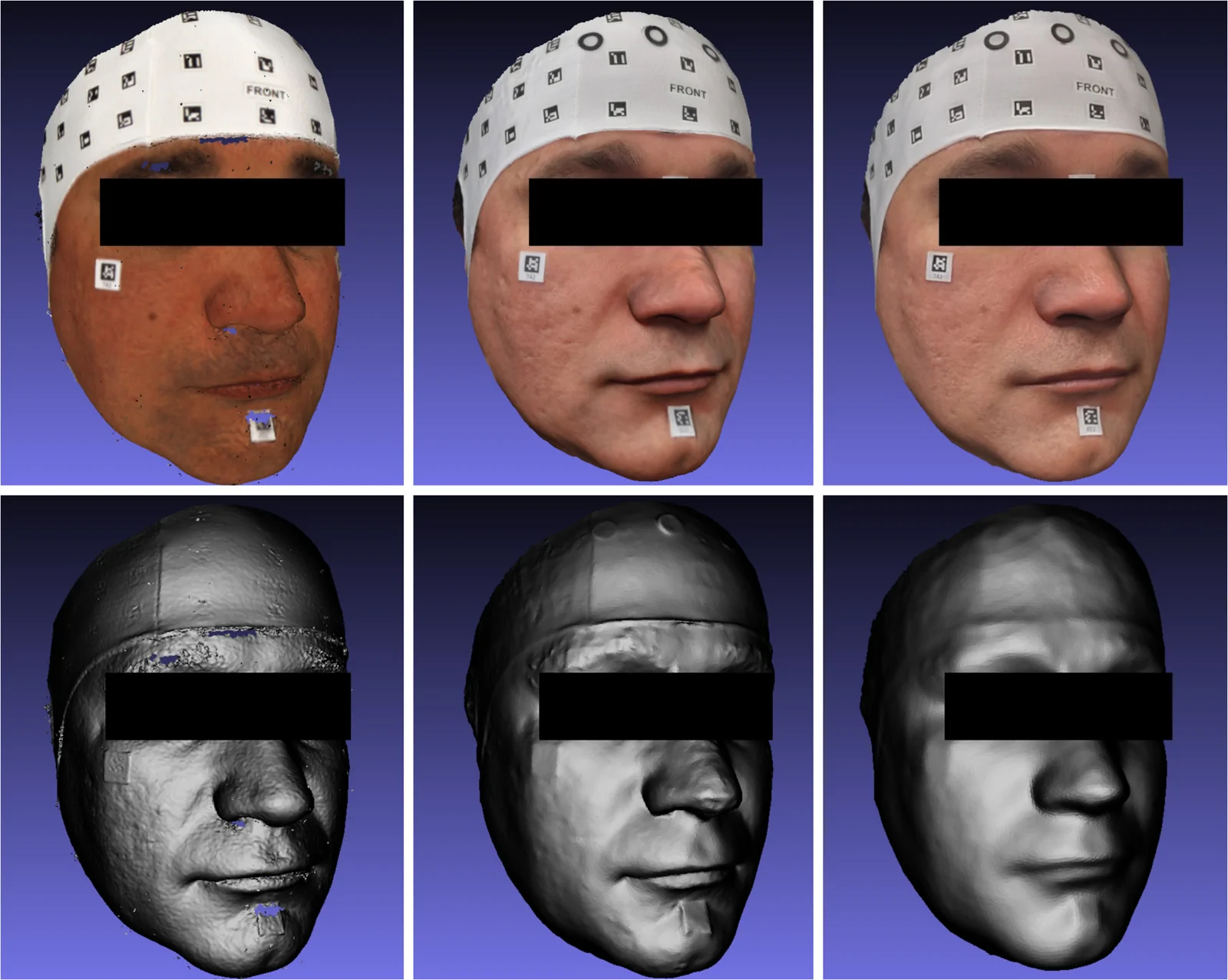
Examples of 3D meshes generated using three acquisition methods: structured light scanning, photogrammetry, and videogrammetry (left to right). Although all methods produce visually similar textured meshes, the shaded renderings highlight differences in geometric resolution: the structured light scan exhibits the highest vertex density, the photogrammetric model shows an intermediate level of detail, and the videogrammetric model has the lowest vertex count. These differences primarily reflect variations in image resolution (video frames versus still photographs) and the inherent precision of the scanning system

This process yielded three models per subject: one from the scanner, one from photos, and one from video (Fig. 2). Each mesh was trimmed to isolate the facial region only. All three meshes were computed according to the same metric system; despite having arbitrary origins, their reference to a common metric scale ensured consistent distance measurements across the 3D models.

**Fig. 3:**
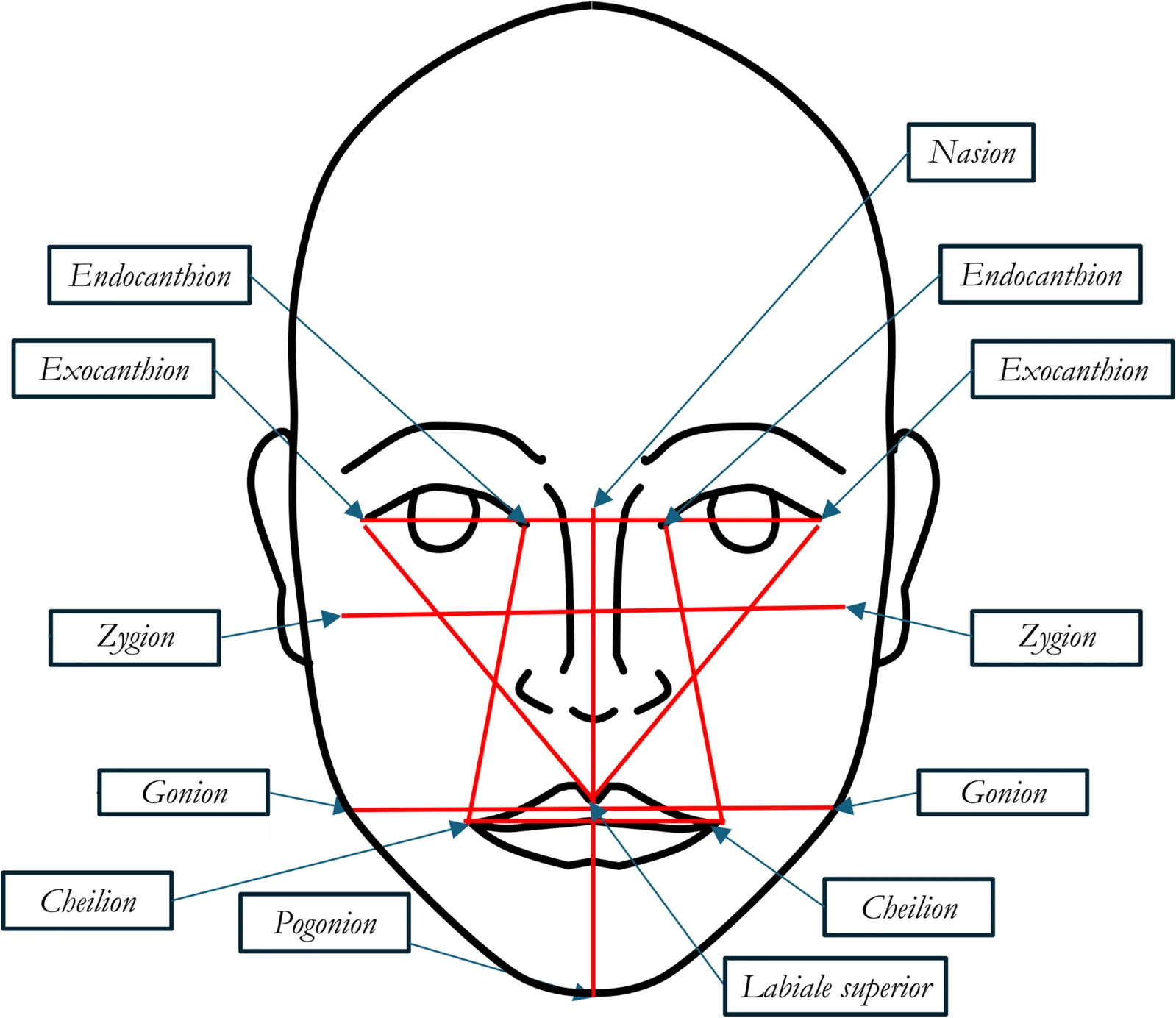
A diagram showing the twelve measured straight-line distances between some facial landmarks, and a few samples of the measurements undertaken on MeshLab

Subsequently, straight-line distances between key facial landmarks were measured on all three models. Landmark selection was adapted from [39] and [40] to align with the sticker placements. The measured distances are shown in Fig. 3.

#### Statistical analysis

The data analysis followed a workflow designed to verify measurement consistency across meshes, ensuring that low-cost photogrammetric solutions are comparable to higher-quality scans. Subsequently, a more qualitative analysis was performed to identify facial landmarks most prone to measurement variation and uncertainty, with the goal of distinguishing subject-specific feature instabilities from methodological inconsistencies.

Initially, a series of parametric, non-parametric, paired and simultaneous hypothesis tests were performed on the full, unfiltered dataset (which included all measurements from all subjects, across all methods). Therefore, raw landmark distance data from each method (photogrammetry, videogrammetry, and structured light scanning) were tested for compatibility - both as pairs and as a set. No outlier-removal procedure was applied, as the small sample size necessitated retaining every observation. Through this, it was possible to identify the most divergent distances across the three acquisition methods, which may be attributable to landmark instability, facial micro-movements, acquisition artefacts, or modality-specific reconstruction limits.

The tests used are described as follows:

- The Kruskal-Wallis test [41], conducted with a significance level of 5%, was used to compare all three datasets simultaneously. This test provides a non-parametric alternative to ANOVA and similar tests, thus assessing if samples originate from the same distribution. Its statistic may be defined as:

1 $$\:H=\frac{12}{N\left(N+1\right)}{\sum\:}_{i=1}^{k}\frac{{R}_{i}^{2}}{{n}_{i}}-3\left(N+1\right)$$

$\:{n}_{i}$: size of sample i

*N*: total sample size

*k*: number of groups being compared

${R}_{i}^{}$: sum of the ranks of the observations in sample i

With the following hypothesis:

${H}_{0}$: the hypothesized measures are not statistically different across the three methods

${H}_{1}$: at least two hypothesized measures are statistically different across the three methods

A *p-*value equal or below 0.05 indicates strong evidence against the null hypothesis, confirming that the measurements differ across the three datasets. A *p*-value above 0.05 suggests insufficient evidence to reject the null hypothesis, meaning no large differences can be detected among the three meshes based on the measurements taken.

Finally, for each set of landmark distances, the three lowest *p*-values were identified to assess the least compatible datasets.

- Additionally, the Wilcoxon–Mann–Whitney [41] and Conover–Iman [42] tests were used for pairwise comparisons among the three methods (photogrammetry vs. videogrammetry, photogrammetry vs. structured light scanning, and videogrammetry vs. structured light scanning). These non-parametric tests were employed to verify whether each pair of datasets could be assumed to originate from the same distribution. For each set of landmark distances, the three lowest *p*-values were also singled out in order to assess the lowest compatibility among datasets. The test statistics for these tests are described as follows:

For the Wilcoxon-Mann-Whitney test:

2 $$\:N={n}_{1}+{n}_{2}$$

3 $${U}_{1}={n}_{1}{n}_{2}+\frac{{n}_{1}\left({n}_{1}+1\right)}{2}-{R}_{1}$$

4 $$\:{U}_{2}={n}_{2}{n}_{1}+\frac{{n}_{2}\left({n}_{2}+1\right)}{2}-{R}_{2}$$

5 $$\:U=\left({U}_{1},{U}_{2}\right)\:$$

Where:

$\:{n}_{1}$, $\:{n}_{2}$: number of observations in samples 1 and 2

$\:{R}_{1}$, $\:{R}_{2}$: sum of the ranks of the observations in samples 1 and 2

For the Conover-Iman test:

6 $$\:t=\frac{\left|\frac{{R}_{1}}{{n}_{1}}-\frac{{R}_{2}}{{n}_{2}}\right|}{\sqrt{\frac{N\left(N+1\right)}{12}\left(\frac{1}{{n}_{1}}+\frac{1}{{n}_{2}}\right)}}$$

Where:

*t*: test statistic comparing samples 1 and 2

$\:{n}_{1}$, $\:{n}_{2}$: number of observations in samples 1 and 2

$\:{R}_{1}$, $\:{R}_{2}$: sum of the ranks of the observations in samples 1 and 2

* N*: total number of observations across all groups

With the following hypothesis:

$\:{H}_{0}$: the hypothesised measures are not statistically different across both methods.

$\:{H}_{1}$: the hypothesised measures are statistically different across both methods.

A *p*-value equal or below 0.05 indicates strong evidence against the null hypothesis, confirming that the measurements differ across each pair of datasets. A *p*-value above 0.05 suggests insufficient evidence to reject the null hypothesis, meaning no large differences can be detected between the two meshes compared, based on the measurements taken.

- The Friedman test [44, 45, 46] is a non-parametric alternative to the repeated-test ANOVA, which is parametric. It is used in order to detect differences in treatments on dependent, repeated-measures data. The test consists of:
  - (1) arranging the data in a matrix *x* with *n* rows (or *blocks*) and *k* columns, where each block is an observation or measurement, and each column *k* stands for a treatment;
  - (2) ranking the values for each *block*, replacing the values of the original *x* matrix with the rank values *r*;
  - (3) finding the mean value of *r* for each column *k;*
  - (4) calculating the Q value using the following formula:
    7 $$\:Q\:=\:\frac{12n}{k(k+1)}{\sum\:}_{j\:=\:i}^{k}{(\underset{\_}{{r}_{j}}-\frac{k+1}{2})}^{2}$$
  - When the number of blocks is greater than 15 and/or the number of treatments is greater than 3, Q values follow a chi-squared distribution. Otherwise, hypothesis tests should follow the Upper Critical Values for the Friendman Test table in order to avoid inaccurate* p*-values.

With the following hypothesis:

$\:{H}_{0}$: the hypothesised measures are not statistically different across all treatments

$\:{H}_{1}$: at least one of the hypothesised measures are statistically different across all treatments

If the null hypothesis is rejected, post-hoc tests should be used to identify which treatments are different of each other.

- The Wilcoxon signed-rank test [46], similarly to the Friedman test, consists of a non-parametric rank test for paired, co-dependent data groups. For each pair of observations:
  1. the difference between both observations is calculated;
  2. the *absolute values of the differences* is ranked;
  3. then the sum of the ranks for all positive and negative differences is computed and called W+ or W-
  4. the Wilcoxon Signed-Rank W value is the smaller between W+ or W-

The hypothesis are:

$\:{H}_{0}$: the paired-differences of both groups are symmetric around a mean value equal to zero

$\:{H}_{1}$: the paired-differences of both groups are symmetric around a mean value not equal to zero

Finally, for each set of landmark distances, the three lowest *p*-values were identified to assess the least compatible datasets.

Following the primary hypothesis tests, which were conducted on the complete, unfiltered dataset, a separate secondary procedure was implemented. This meant to flag potential outliers. A Shapiro-Wilk test was iteratively reapplied by removing, at each cycle, the most extreme observation until the remaining sample satisfied the normality assumption. Importantly, this iterative exclusion served as a secondary diagnostic and did not affect the initial inferential statistics. It provided another insight for identifying the most divergent distances across the three acquisition methods.

This additional procedure was implemented according to the following steps:

- Raw landmark distance data were normalised using multiple approaches to ensure comparability and mitigate systematic biases inherent to each 3D reconstruction method. More specifically, sample sets were created in which the data from each method was normalised against data from the other two methods. This technique serves as cross-validation and helps isolate true physiological instability from artefacts derived from different methodologies.
- Finally, one procedure was conducted in order to verify both landmark distances that stand out as outliers. This routine was implemented in Python and iteratively devised. In each iteration, the most evident outlier was detected using the Interquartile Range (IQR) test, which subtracts 1.5 times the IQR from the first quartile (Q1) and adds it to the third quartile (Q3). After removing the most evident outlier, the sample was checked for normality using the Shapiro-Wilk test with a significance level of 5%, which tests if the sample comes from a normally-distributed population according to the methodology originally developed by [43], as would be expected from an independent group of patients. Each iteration, therefore, removed one outlier and assessed if the remaining data converged towards a normal distribution. The process continued running the same routine until the sample passed the Shapiro-Wilk test, after which it would stop and produce a report listing both patients and landmark distances found to be inconsistent. Finally, tables were used to compare the variability of key landmark distances across methods, illustrating the distribution and dispersion of measurements. Disagreement was quantified as a percentage variation for each critical landmark, and landmark distances were subsequently ranked by reliability with associated uncertainties.

## Results

### Absolute inter-landmark distances statistics

Table 2 summarizes basic statistics such as mean, standard deviation, median, range, and mean (in millimeters) for all relevant landmark distances and methods.

**Table 2:** Absolute inter-landmark distances descriptive statistics, in millimeters (mm)

Measurement	Mean	Median	Standard Deviation	Max	Min	Range
Cheilion-Cheilion	48.14	49.50	4.77	55.00	33.00	22.00
Cheilion-Endocanthion (left)	68.38	66.70	5.11	78.50	60.70	17.80
Cheilion-Endocanthion (right)	69.00	68.20	5.48	78.00	59.50	18.50
Exocanthion-Exocanthion	90.98	89.30	6.25	108.40	80.40	28.00
Gonion-Gonion	124.18	121.40	10.62	146.40	106.40	40.00
Labiale superior - Exocanthion (left)	79.00	79.10	5.94	88.20	60.40	27.80
Labiale superior - Exocanthion (right)	79.58	77.80	6.10	89.10	62.60	26.50
Nasion-Pogonion	96.09	94.40	7.24	112.00	85.40	26.60
Zygion-Zygion	113.98	114.00	8.05	133.20	99.40	33.80

### Hypothesis tests on landmark distances across the three methods

As previously explained, distances in centimeters were computed for each measurement of key landmark distances, for each one of the fifteen subjects. These were subject to three hypothesis tests: a Kruskal-Wallis test comprising the three datasets at once, and both a Wilcoxon-Mann-Whitney test and a Conover-Iman test on pairs of data acquired through different methods: (photogrammetry vs. videogrammetry, photogrammetry vs. structured light scanning, and videogrammetry vs. structured light scanning).

Table 3 summarizes the results of the Kruskal-Wallis test. All *p*-values are considerably above the 0.05 significance level, indicating that the null hypothesis cannot be rejected. Therefore, no statistically significant differences are observed across the three methods.

**Table 3:**
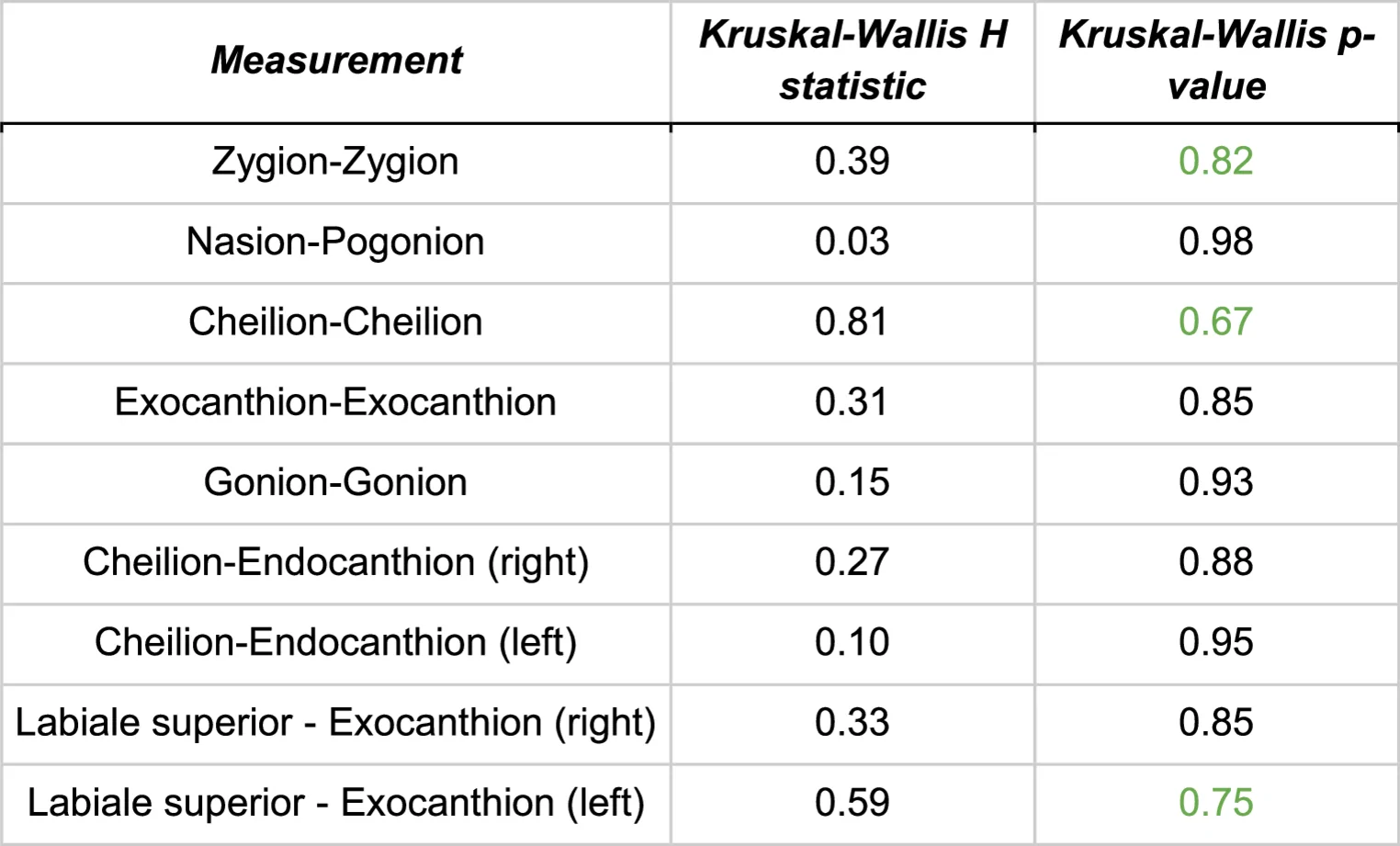
Summary of Kruskal-Wallis F statistics and respective *p*-values for the measured distances, when comparing the three methods at once. Values in green indicate the lowest *p*-values

However, despite overall statistical consistency among the methods, certain distances yielded notably lower *p*-values. The three lowest correspond to Cheilion–Cheilion, Zygion–Zygion, and Labiale superior–Exocanthion (left). While these values remain above the significance threshold, they suggest relatively greater divergence between methods for these specific landmarks—warranting closer examination in future analyses.

Table 4 summarizes the results of the Wilcoxon-Mann-Whitney test. As with the Kruskal-Wallis test, all *p*-values are considerably above the 0.05 significance level, indicating that the null hypothesis cannot be rejected. Therefore, no statistically significant differences are observed across methods when compared in pairs.

**Table 4:**
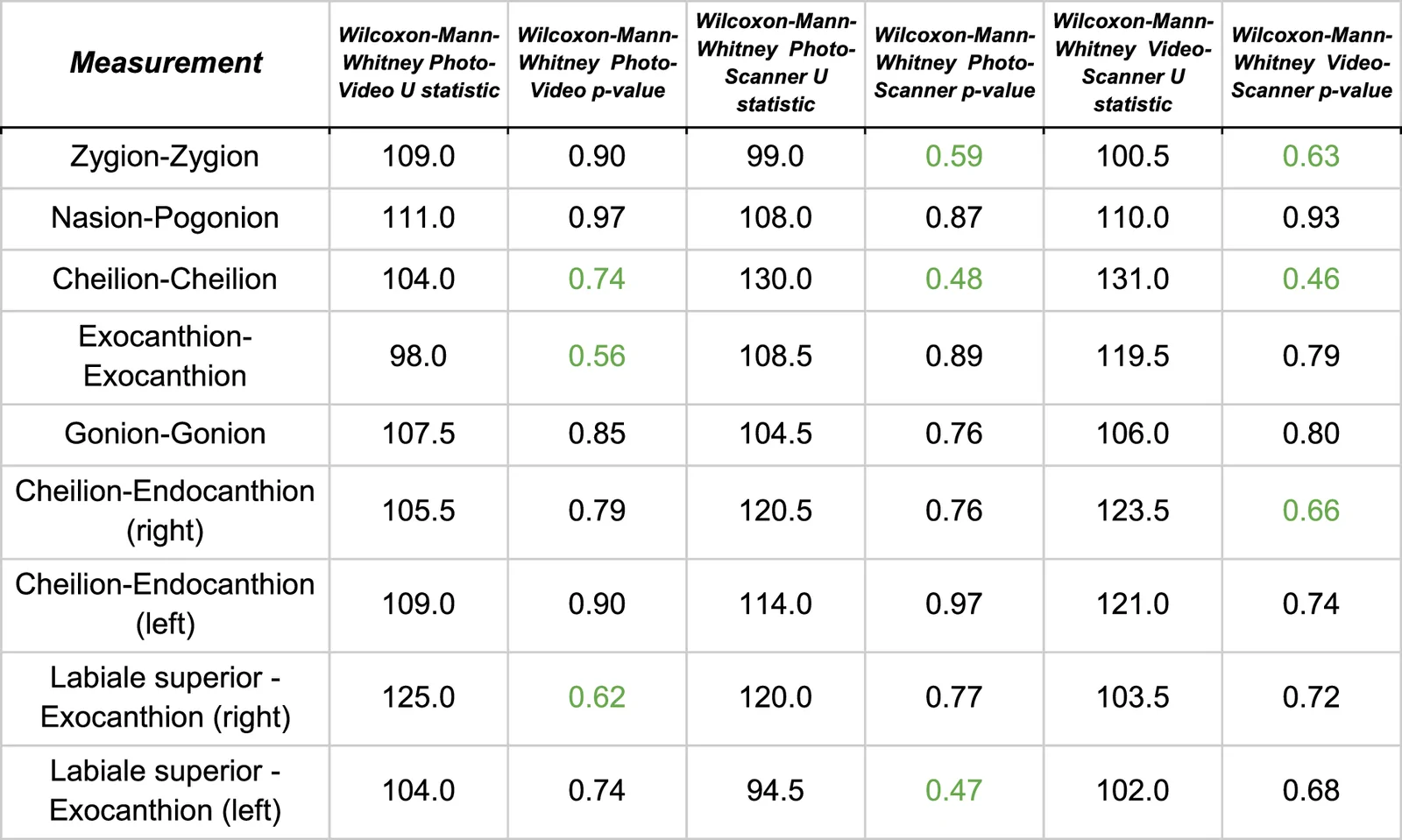
Summary of Wilcoxon-Mann-Whitney statistics and respective *p*-values for the measured distances, when comparing the methods by pairs. Values in green indicate the lowest *p*-values

The pairwise analysis of *p*-values reveals several trends. First, *p*-values are consistently higher when comparing distances measured from photogrammetric and videogrammetric meshes, suggesting these two models are more consistent with each other. This may be due to the fact that both were acquired using the same phone, despite the sensors are different and cannot be considered stable photogrammetric devices.

Among the three lowest *p*-values for each pair of methods, Cheilion–Cheilion appears in all three pairs, highlighting it as the most recurrent distance in terms of divergence. Zygion–Zygion appears in two pairs, while Cheilion–Endocanthion (right), Labiale superior–Exocanthion (right), and Labiale superior–Exocanthion (left) each appear once.

Table 5 summarizes the results of the Kruskal-Wallis test. As with previous tests, all *p*-values are considerably above the 0.05 significance level, indicating that the null hypothesis cannot be rejected. Therefore, no statistically significant differences are observed across methods when compared in pairs.

**Table 5:**
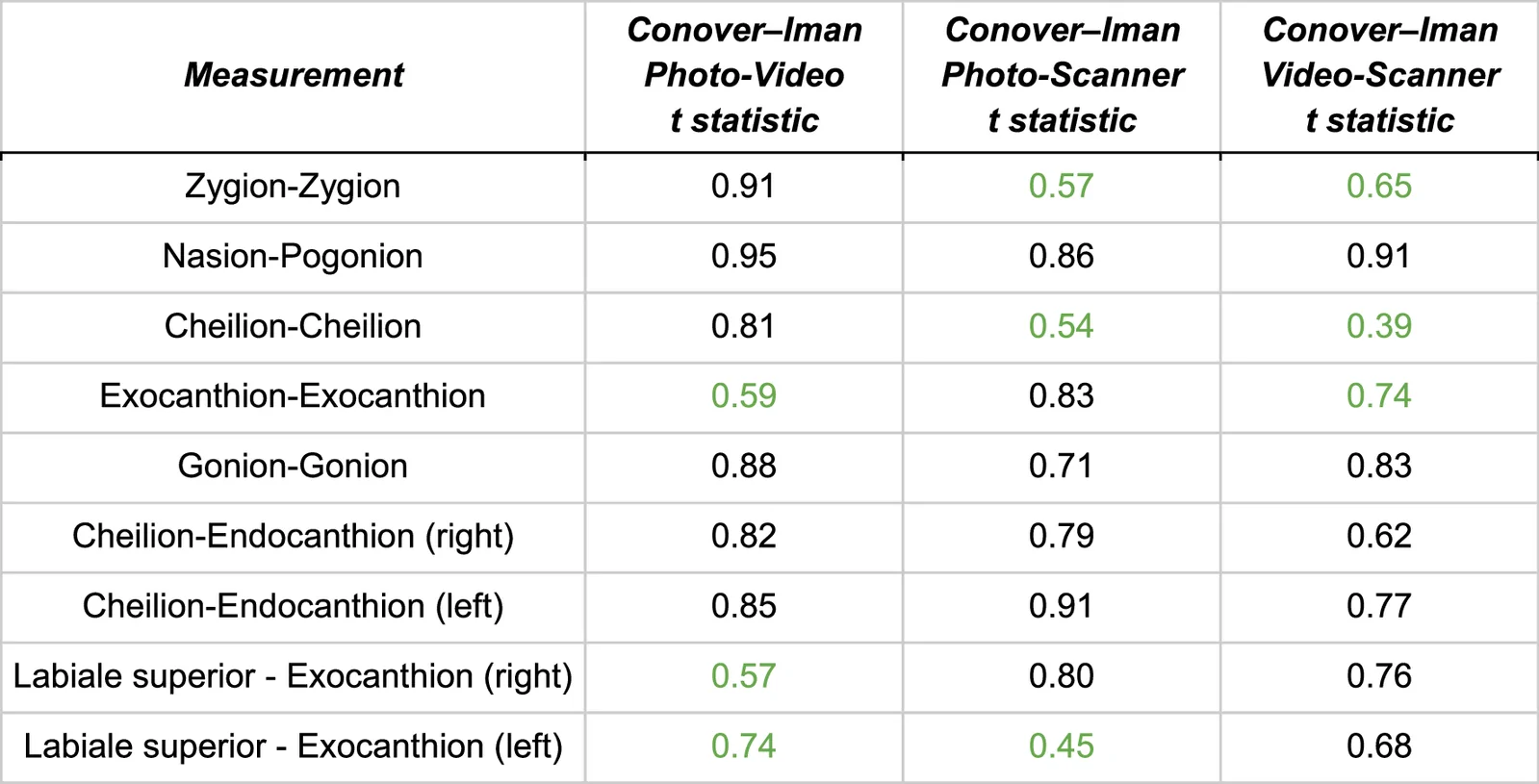
Summary of Conover–Iman *p*-values for the measured distances, when comparing the methods by pairs. Values in green indicate the lowest *p*-values

The pairwise analysis of *p*-values reveals trends consistent with those observed in the Wilcoxon–Mann–Whitney test. Although the *p*-values for comparisons between photogrammetric and videogrammetric meshes are not substantially higher than those for the other pairwise comparisons, they remain comparatively higher. Among the three lowest *p*-values across pairs, Cheilion–Cheilion, Zygion–Zygion, Exocanthion–Exocanthion, and Labiale superior–Exocanthion (left) each appear in two pairs, while Labiale superior–Exocanthion (right) appears in only one.

Tables 6 and 7 provide a summary of Friedman Q and *p*-values, alongside Conover-Iman post-hoc values for the measured distances, compared in pairs. Such results also corroborate trends akin to previous tests. Even if all landmark distances pass the test, Cheilion-Endocanthion (right), Cheilion-Cheilion and Zygion-Zygion consistently show lower results in terms of *p*-values.

**Table 6:**
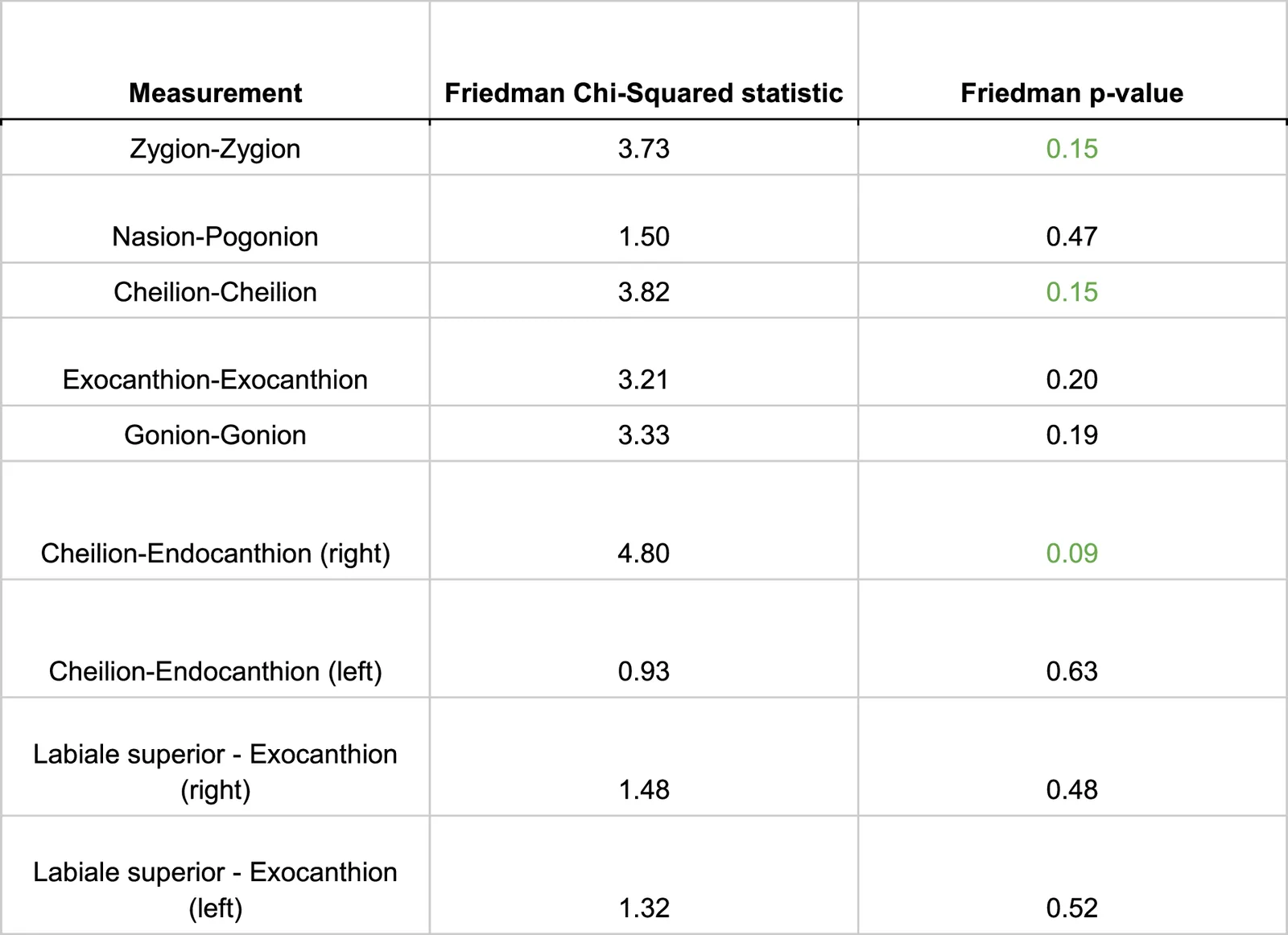
Summary of Friedman Q and *p*-values for the measured distances, when comparing the three methods at once. Values in green indicate the lowest *p*-values

**Table 7:**
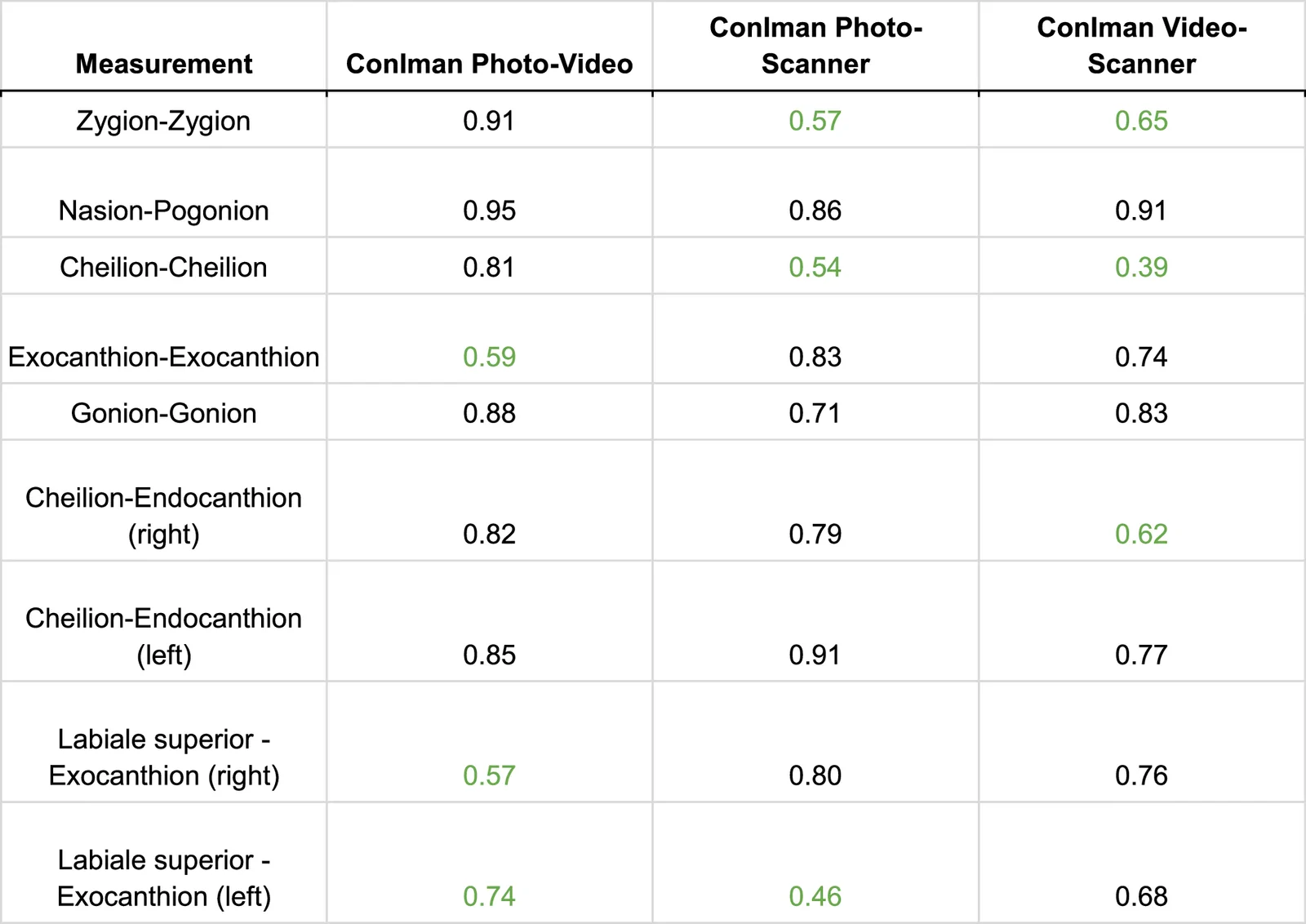
Summary of Conover–Iman post-hoc *p*-values for the measured distances, comparing the methods by pairs. Values in green indicate the lowest *p*-values

Finally, Table 8 provides a summary of results for the Wilcoxson signed-rank test. This is the only test where a few distances had the null hypothesis rejected. These were: Zygion-Zygion and Cheilion-Cheilion (for photo-scanner), Exocanthion-Exocanthion (for photo-video), and Cheilion-Endocanthion (right) (for video-scanner).

**Table 8:**
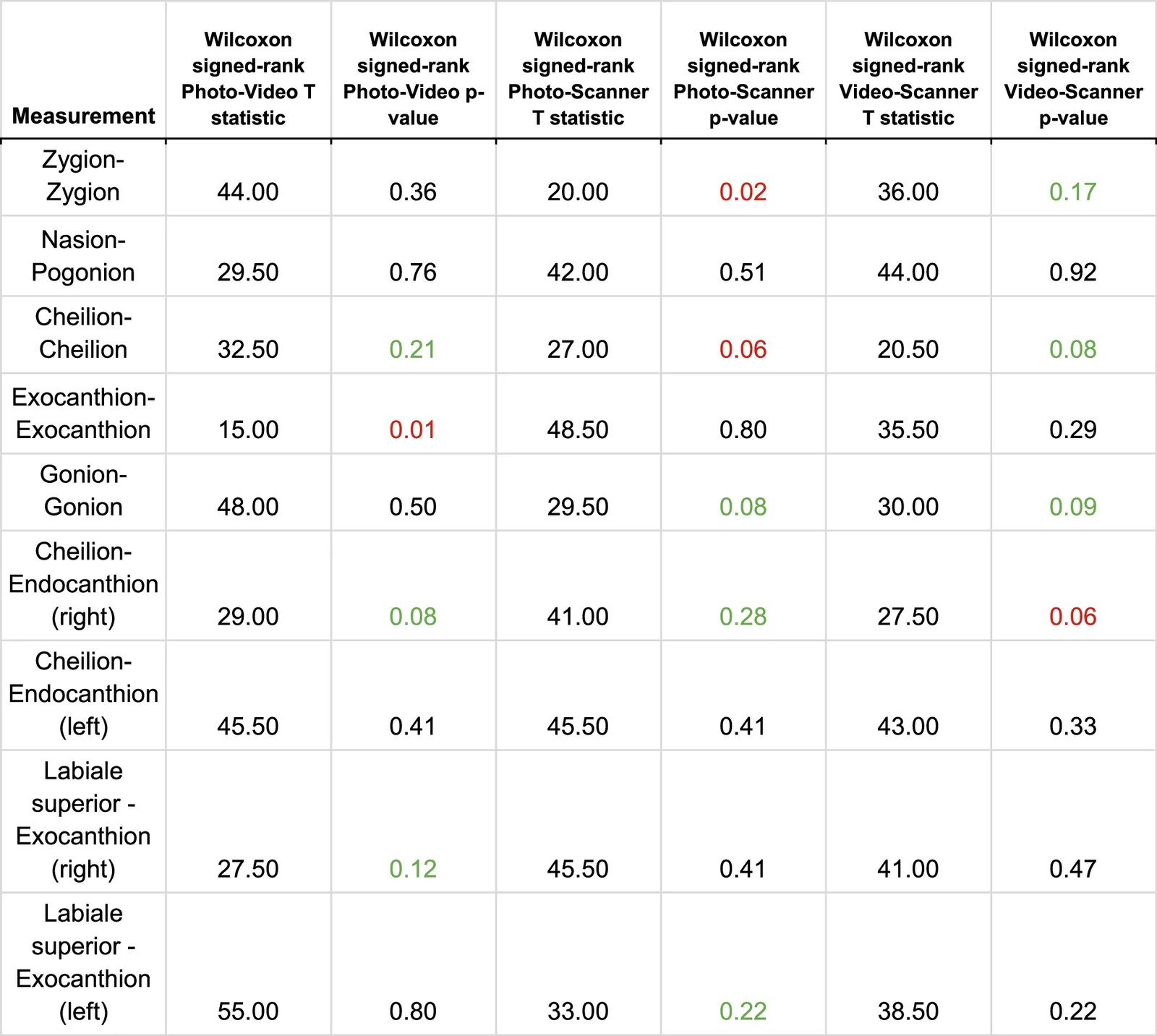
Summary of Wilcoxon signed-rank sum of the ranks sum and *p*-values for the measured distances, comparing the methods by pairs. Values in green indicate the lowest *p*-values and in red indicate rejection of H0 and the acceptance of the two-sided alternative hypothesis

In total, the following landmark distances rank among the lowest *p*-values (or had the null hypothesis rejected in the Wilcoxon signed-rank test: Cheilion-Cheilion (eight out of nine times), Zygion-Zygion (seven out of nine times), Labiale superior–Exocanthion (left) (four out of nine times), Exocanthion-Exocanthion (four out of nine times), Cheilion–Endocanthion (right) (three out of nine times) and Labiale superior–Exocanthion (left) (two out of nine times).

### Landmark distances assessment

Following the hypothesis test, a cross-normalisation of the three measurement sets was conducted, thus generating six distinct datasets. These correspond to each pairwise Method/Normalisation combination: (a) Photogrammetry/Videogrammetry, (b) Photogrammetry/Scanner, (c) Videogrammetry/Photogrammetry, (d) Videogrammetry/Scanner, (e) Scanner/Photogrammetry, and (f) Scanner/Videogrammetry. This reciprocal approach (where, for example, Photogrammetry is normalised by Videogrammetry and vice versa) was intentional. It ensured that no important information was lost due to the change in metrics. For the Shapiro-Wilk tests, the null hypothesis was that the data followed a normal distribution. The value of the W statistic was 0.858 for a *p*-value less or equal than 0.05. Initially, the null hypothesis was rejected for all six different datasets.

The initial outlier testing was also performed for all landmark distances in all patients. Table 9 summarizes the steps taken to normalize each combination dataset from (a) to (f).

**Table 9:** Iterative process for the whole 15-patient combination dataset, listing which landmark distances showed patient outliers and their magnitude

Dataset	Iteration	Worst outlier	Number of landmark outliers	Difference (%) for outliers	Mean & standard-deviation for non-outliers
Photo/Video (a)	1	Labiale superior - Exocanthion (left)	3	Patient 1: 9.05 Patient 4: 19.24 Patient 14: 22.52	1.41 ± 1.10
	2	Labiale superior - Exocanthion (right)	1	Patient 14: 21.57	1.39 ± 1.04
	3	Cheilion-Cheilion	1	Patient 11: 15.15	1.32 ± 1.01
Photo/Scanner (b)	1 2	Labiale superior - Exocanthion (left) Cheilion-Cheilion	3 3	Patient 4: 20.75 Patient 8: 5.61 Patient 15: 6.22 Patient 7: 5.66 Patient 9: 6.43 Patient 11: 7.65	1.52 ± 1.26 1.45 ± 1.21
	3	Exocanthion-Exocanthion	2	Patient 4: 9.87 Patient 8: 6.73	1.38 ± 1.14
	4	Labiale superior - Exocanthion (right)	2	Patient 4: 5.49 Patient 7: 6.87	1.37 ± 1.08
	5	Cheilion-Endocanthion (right)	1	Patient 8: 7.47	1.29 ± 1.06
Video/Photo (c)	1	Labiale superior - Exocanthion (left)	3	Patient 1: 8.30 Patient 4: 23.82 Patient 14: 18.38	1.43 ± 1.14
	2	Labiale superior - Exocanthion (right)	1	Patient 14: 17.74	1.41 ± 1.12
	3	Cheilion-Cheilion	1	Patient 11: 13.16	1.33 ± 1.05
Video/Scanner (d)	1	Cheilion-Cheilion	4	Patient 3: 8.63 Patient 9: 8.71 Patient 11: 6.52 Patient 13: 9.20	1.69 ± 1.43
	2	Labiale superior - Exocanthion (left)	2	Patient 1: 12.61 Patient 14: 16.34	1.67 ± 1.39
	3	Labiale superior - Exocanthion (right)	1	Patient 14: 16.42	1.66 ± 1.37
Scanner/Photo (e)	1 2	Labiale superior - Exocanthion (left) Cheilion-Cheilion	3 3	Patient 4: 26.18 Patient 8: 5.95 Patient 15: 6.63 Patient 7: 5.35 Patient 9: 6.04 Patient 11: 7.11	1.45 ± 1.16 1.37 ± 1.09
	3	Exocanthion-Exocanthion	2	Patient 4: 10.95	1.29 ± 1.02
	4	Labiale superior - Exocanthion (right)	2	Patient 4: 5.80 Patient 7: 6.43	1.26 ± 0.99
	5	Cheilion-Endocanthion (right)	1	Patient 8: 8.07	1.16 ± 0.94
Scanner/Video (f)	1 2	Cheilion-Cheilion Labiale superior - Exocanthion (left)	4 2	Patient 3: 7.95 Patient 9: 8.02 Patient 11: 6.97 Patient 13: 8.42 Patient 1: 14.43 Patient 14: 19.54	1.61 ± 1.32 1.60 ± 1.30
	3	Labiale superior - Exocanthion (right)	2	Patient 4: 5.68 Patient 14: 19.65	1.59 ± 1.29
	4	Cheilion-Endocanthion (right)	1	Patient 8: 7.53	1.51 ± 1.25

Similarly to the first table, dataset (a) Photogrammetry/Videogrammetry in iteration 1 showed that the landmark distance Labiale superior–Exocanthion (left) was an outlier in three patients: Patient 1, with a 9.05% percentage difference between methods; Patient 4, with 19.24%; and Patient 14, with 22.52%. All other landmark distances had a mean of 1.41% ± 1.10%.

The three landmark distances for Labiale superior–Exocanthion (left) were then removed. After testing for normality, another landmark distance (Labiale superior–Exocanthion (right)) was identified as the worst outlier, with a 21.57% difference for Patient 14. As the data still failed the normality test, this outlier was removed. The procedure was repeated, revealing Cheilion–Cheilion as the new worst outlier with a 15.15% difference in the landmark distance for Patient 11. Following its removal, the dataset from all subjects passed the normality test.

**Fig. 4:**
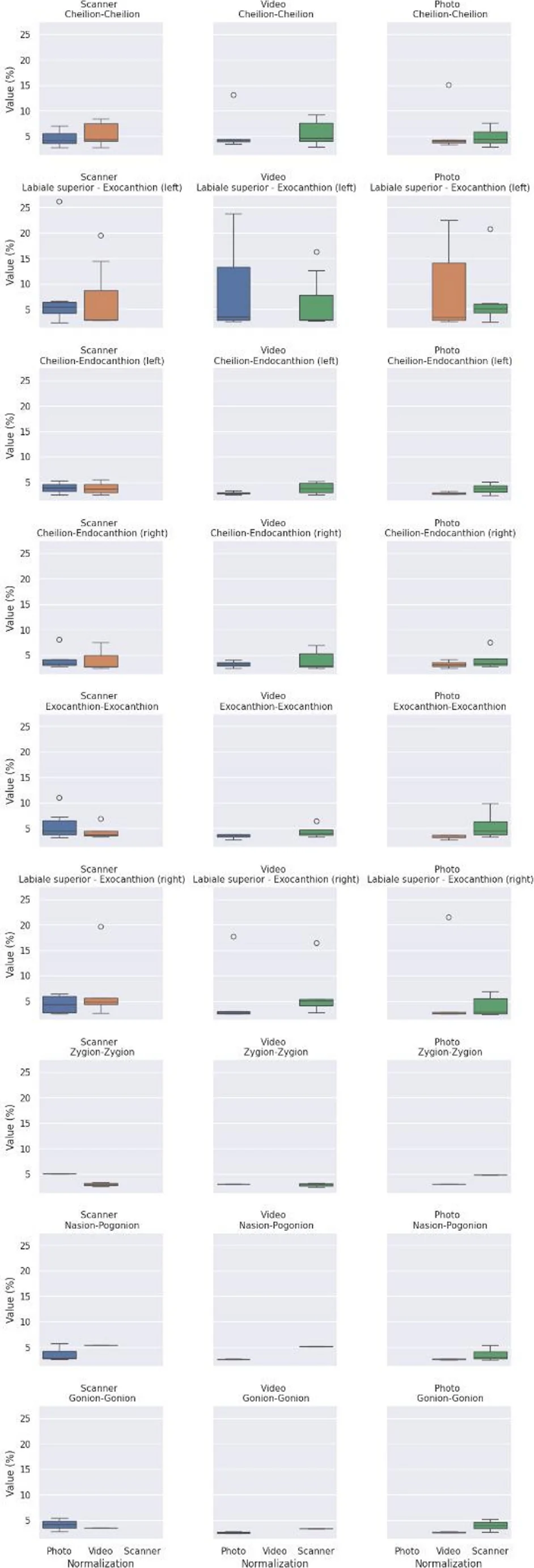
Box-and-whiskers diagrams showing percentage variations across multiple data acquisition and normalization methods

The remainder of Table 9 details the analogous steps required to achieve normality in the other combination datasets. A noteworthy pattern emerged regarding the most frequent outlier landmark distances: Cheilion–Cheilion and Labiale superior–Exocanthion (left) were singled out 14 times each, Labiale superior–Exocanthion (right) 8 times, Exocanthion–Exocanthion 6 times, Cheilion–Endocanthion (right) 3 times, Nasion–Pogonion 2 times, and Gonion–Gonion 1 time. Figure 4 illustrates that this discrepancy persists across multiple acquisition methods and normalization techniques. In particular, the measurements for Labiale superior–Exocanthion (left) and Cheilion–Cheilion show especially pronounced discrepancies.

## Discussion

These results allow us to confirm the promising usage of low-cost 3D imaging and 3D models for detecting facial asymmetry. A clear convergence was observed between the outputs of low-cost image-based and active optical 3D reconstruction technologies, as hypothesis tests suggest. Based on the results of the three tests, it is reasonable to conclude that all the evaluated methods produce highly similar 3D models [15, 27], which serve as accurate representations of facial topography suitable for a wide range of applications [47]. This consistency supports previous research validating the clinical accuracy of standard photogrammetry and videogrammetry, even compared with high-resolution white light scanners [17, 27], but extends it to low-cost imagery not yet extensively used for medical applications.

However, it is important to distinguish between statistical similarity and practical interchangeability. Although the three technologies produced highly comparable measurements and generated 3D models with similar geometric characteristics, the absence of statistically significant differences alone does not demonstrate that the methods are fully interchangeable in all contexts. Interchangeability depends on whether the observed measurement discrepancies remain within an application-specific tolerance threshold. In the present study, the majority of landmark distances had minor differences, and this suggests a high degree of agreement for most facial regions. However, larger discrepancies observed in specific landmarks indicate that caution is warranted when considering the replacement of structured-light scanning by smartphone-based photogrammetry or videogrammetry in applications requiring higher precision. Future studies should establish clinically meaningful tolerance limits in millimetres and evaluate agreement against those predefined thresholds.

Another important observation is that, while the technical accuracy of short-range 3D reconstruction is influenced by factors such as image resolution, geometric configuration, lens distortion, and illumination, a critical and often overlooked challenge in human subject research is the inherent non-rigidity and instability of the human face. This study has not considered any human error involving landmark positioning due to anatomical constraints (e.g. zygon, gonion, etc.), but reflects how anatomical areas affected by uncertainty in positioning (mandibular & zygomatic areas) and dynamic facial movements (Cheilion, Labiale superior) are less reliable for high-quality assessment.

Involuntary facial movements (even subtle ones) can compromise the reliability of measurements, particularly for landmarks located in mobile landmarks, such as those affecting the lips (Cheilion and Labiale superior). This is an important consideration when using low-cost photogrammetric methods, which depend on the subject being as still as possible while photos (or video frames) are taken. Our findings indicate that measurements involving the perioral region–specifically the Cheilion and Labiale Superior landmarks–are considerably less precise than all other measurements taken from the face. The level of uncertainty for these facial landmarks was consistently around ± 10%, as opposed to other landmark distances, which showed differences of less than ± 3% on average. This suggests that perioral landmarks may be suboptimal for use as reliable facial landmarks. If their use is necessary, it must be undertaken with a clear acknowledgement of this inherent uncertainty and a tentative margin of error of at least ± 10% (based on our analysis).

Likewise, worth noticing is that facial landmarks in the orbital region, which have traditionally been considered in medical literature [38], are not as reliable as expected. In particular, the uncertainty with the Exocanthion (both left and right) is higher than with the Endocanthion, which reinforces the premise that, for a landmark to be reliable, it must be sufficiently static and independent of involuntary movement.

These discrepancies observed in measurements involving the Labiale Superior, Cheilion, and Exocanthion landmarks may be interpreted as a central finding, since they share anatomical and technical characteristics that may contribute to increased uncertainty. The perioral region is highly susceptible to involuntary muscular activity, subtle variations in lip posture, and also in facial tension. These may effectively alter landmark position between acquisitions. Similarly, the Exocanthion is located at the lateral commissure of the eyelids, where soft-tissue definition is less pronounced and where small variations in eye opening, blinking, or facial expression may affect landmark identification. And last, but not least, these regions often exhibit more complex local surface geometry, potentially increasing reconstruction uncertainty in image-based methods.

These findings suggest that certain facial landmarks exhibit inherent instability (which yields to an inherent uncertainty) in live subjects and that such variability might stem not only from measurement techniques but also from behavioural factors such as involuntary facial movements, muscle tension, and inconsistent head positioning. These factors introduce uncertainty in facial landmark positioning, particularly in dynamic areas. Moreover, landmarks on bony or structurally stable regions, typically considered to yield more consistent and reproducible measurements, as suggested by [28], have been proven to be reliable as long as those landmarks are easily identifiable and are not subject to involuntary movement. The consistency of these discrepancies across technologies suggests that landmark instability may be an intrinsic characteristic of these anatomical regions rather than a limitation of any single acquisition technique. Consequently, the identification of reliable and unreliable landmarks should be regarded as one of the principal outcomes of this study.

Another critical factor to consider is the pervasive reliance on manual landmark selection in current research methodologies. A survey of the relevant literature reveals that in the vast majority of studies utilising 3D models (either from photogrammetry or 3D scanning), the actual process of identifying landmarks and performing anthropometric measurements is conducted manually by healthcare professionals or researchers. This method depends entirely on the operator’s visual interpretation of the model’s geometry and surface textures. Whether the landmarks are physical markers placed on the subject (which can shift or be positioned in low-contrast areas) or natural, requiring high-contrast anatomical features, this manual approach introduces an inherent and unquantifiable layer of uncertainty and inter-observer variability. This study aimed to follow the measurement steps in accordance with current research in the field. However, this added layer of uncertainty underscores a critical future step: the development and implementation of automated landmarking algorithms, potentially employing established libraries for facial recognition and analysis.

An open question is whether anatomical points such as the Tragus and the Pronasale can eventually be analysed as reliable facial landmarks because they meet the criteria of being easily identifiable and less prone to inter-observer error; more importantly, they are less sensitive to movement.

While 3D scanning, photogrammetry, and videogrammetry are reliable for measuring facial asymmetry, a critical challenge persists: it is difficult to determine whether measurement errors originate from the methodologies themselves or from subtle, unconscious movements of the human face that introduce slight variations across acquisitions and technologies. Identifying which landmarks contribute to determining robust asymmetry indices is therefore essential to improve diagnostic accuracy and ensure reproducibility in both research and clinical practice. This distinction is paramount, as the growing reliance on 3D models means such variations directly compromise the accuracy of landmark positioning. To address this, our study isolated these factors by generating a dataset of forty-five 3D facial meshes from fifteen patients using three different technologies (3D scanning, photogrammetry, and videogrammetry).

Therefore, from a practical perspective, the results support the use - under certain scenarios - of smartphone-based photogrammetry and videogrammetry for facial documentation, anthropometric studies, longitudinal monitoring, educational purposes, and large-scale data collection. The high level of agreement observed with structured-light scanning indicates that these approaches can provide sufficiently accurate representations of facial morphology for some research and clinical applications. Nevertheless, applications requiring the highest levels of geometric precision (such as some kinds of surgical planning and postoperative assessments that require milimetric changes, or even some kinds of high-precision forensic identification) need to be approached with greater caution. In such contexts, the uncertainty associated with specific landmarks and the inherent variability of facial soft tissues may require the use of dedicated high-precision scanning systems and stricter validation protocols.

Finally, the development and testing of PhotoMeDAS have demonstrated that the strategic placement of additional markers is an effective enhancement for extending measurement coverage beyond the cranial region. Studies such as this one have helped identify the need for and support the incorporation of supplementary markers to improve the capture of anatomical areas not adequately represented by the original configuration. As of now, PhotoMeDAS has evolved into several versions tailored to different clinical and research applications: the Head Cap (Version AT), designed exclusively for cranial measurements; the Orthotic Helmet Cap (Version OR), which extends coverage to the lateral regions of the head; and the Complete Head and Face Cap (Version CA), which incorporates eight manually placed coded markers to enable comprehensive documentation of both cranial and facial structures.

## Conclusion

This study set out to investigate whether low-cost smartphone-based photogrammetry and videogrammetry can achieve measurements comparable to those obtained from structured-light scanning, a current gold standard in clinical 3D imaging. A core challenge was distinguishing measurement variability arising from the acquisition methodologies from that introduced by subtle, unconscious or involuntary facial movements: a distinction critical for high-precision applications such as asymmetry assessment.

Through a multi-stage statistical workflow incorporating non-parametric hypothesis tests, iterative outlier detection, and normality testing, the analysis precisely identified that the three modalities yielded comparable results overall, supporting the adoption of low-cost smartphone-based techniques for facial measurements. However, the primary source of measurement uncertainty was traced to involuntary facial movements, with landmarks in the mobile perioral region (Cheilion and Labiale superior) and Exocanthion proving significantly less precise than more stable landmarks.

Based on these findings, the study concludes that while structured-light scanning, photogrammetry, and videogrammetry are all viable for assessing facial asymmetry, their accuracy is ultimately constrained by the inherent instability of the human face and the limitations of manual landmark placement. To ensure high-precision outcomes, measurements relying on the perioral region should be interpreted with caution or avoided when possible. Future development should focus on adopting automatic landmarking algorithms to eliminate variability introduced by manual annotation, and study designs that incorporate modality-specific considerations to enhance the reliability of facial anthropometric research.

## Data Availability

The datasets used and/or analysed during the current study are available from the corresponding author on reasonable request.
